# Updates on the Pivotal Roles of Mitochondria in Urothelial Carcinoma

**DOI:** 10.3390/biomedicines10102453

**Published:** 2022-10-01

**Authors:** Chiang-Chi Huang, Hui-Ying Liu, Tsuen-Wei Hsu, Wen-Chin Lee

**Affiliations:** 1 Division of Nephrology, Department of Internal Medicine, Kaohsiung Chang Gung Memorial Hospital and Chang Gung University College of Medicine, Kaohsiung 83301, Taiwan; 2 Department of Urology, Kaohsiung Chang Gung Memorial Hospital and Chang Gung University College of Medicine, Kaohsiung 83301, Taiwan

**Keywords:** mitochondrial metabolism, redox homeostasis, apoptosis, tumorigenesis, urothelial carcinoma

## Abstract

Mitochondria are important organelles responsible for energy production, redox homeostasis, oncogenic signaling, cell death, and apoptosis. Deregulated mitochondrial metabolism and biogenesis are often observed during cancer development and progression. Reports have described the crucial roles of mitochondria in urothelial carcinoma (UC), which is a major global health challenge. This review focuses on research advances in the role of mitochondria in UC. Here, we discuss the pathogenic roles of mitochondria in UC and update the mitochondria-targeted therapies. We aim to offer a better understanding of the mitochondria-modulated pathogenesis of UC and hope that this review will allow the development of novel mitochondria-targeted therapies.

## 1. Introduction

Urothelial carcinoma (UC) is a malignancy of the urinary system lining. The majority of UC cases (approximately 90–95%) arise in the urinary bladder. The remaining 5–10% are upper tract urothelial carcinomas (UTUCs), which refer to malignancies that originate from the renal calyceal system to the distal ureter [1,2]. Bladder cancer is indeed a major health threat, with an estimated 573,278 incident cases and 158,785 deaths worldwide in 2020 [3]. Although approximately 75% of newly diagnosed cases are non-muscle-invasive, 70% of tumors will recur, and 20% of the recurrences will progress to muscle-invasive disease that carries a high risk of tumor progression or metastasis. The 5-year survival rate of patients with metastatic disease is only 5% [4,5,6]. UTUC and bladder cancer are biologically similar and possess certain common risk factors, such as smoking and occupational exposure. However, they represent distinct entities owing to anatomical and practical differences [1,7,8,9]. The overall 5-year survival rate for UTUC is approximately 59–67% [10,11] and has been decreasing in recent years [12,13]. Depending on the type of UC and the stage of the disease, the mainstay of treatment includes surgery, radiation therapy, chemotherapy, and immunotherapy. Despite the great progress made in the diagnosis and treatment of UC, especially the rapid advances in immunotherapy, targeted therapy, and combinations [4,14,15,16], the high recurrence and mortality rates indicate that there are unmet needs in the management of UC. Revisiting the pathogenesis of UC may be a solution to the current bottleneck.

Mitochondria are important organelles responsible for energy production, redox homeostasis, oncogenic signaling, cell death, and apoptosis. Mitochondrial metabolism comprises pathways that generate adenosine triphosphate (ATP) and produce components necessary for macromolecule biosynthesis. It has become clear that mitochondrial metabolism plays an influential role in governing cell fate and function by controlling gene expression through the release of metabolites and reactive oxygen species (ROS) [17]. The compartmentalization of mitochondrial protein complexes and enzymes is essential for the maintenance of signaling pathways within the cell. The kidneys are second only to the heart in terms of mitochondrial abundance. In addition to their crucial roles in renal physiology, mitochondria have been recognized as key participants in kidney cancers [18,19,20]. Reduced mitochondrial DNA (mtDNA) content has been observed in renal cell carcinoma (RCC) [21]. In addition, impaired mitochondrial respiratory capacity has been observed in clear cell RCC [22]. Altered mitochondria-regulated apoptotic pathways have been reported in UTUC [23,24].

This review focuses on research advances in the role of mitochondria in UC. We aimed to offer a better understanding of the link between mitochondria and the pathogenesis of UC. We hope that this review will facilitate the development of novel mitochondria-targeted therapies for UC.

## 2. Roles of Mitochondria in UC

### 2.1. Alterations of mtDNA in UC

MtDNA mutations tend to be induced by oxidative damage, defects in nuclear genes in mtDNA stability and replication, altered nucleotide biosynthesis or transport, and exogenous sources (e.g., tobacco smoking, ionizing radiation, ozone, pesticides, and heavy metals) [25,26]. mtDNA mutations have been identified in 64% of bladder cancers and demonstrated in cancer tissue in the form of single-base deletions, point mutations, and insertions in the non-coding D-loop region or deletions in the coding regions of proteins involved in oxidative phosphorylation [27]. For example, among the mitochondrial genes cytochrome B, ATPase6, ND1, and the D310 region, G14905A, G8697A, C15452A, and A15607G polymorphisms were reported to be more frequent in UC patients than in controls [28]. The tumorigenic role of mtDNA mutations in UC was demonstrated for the 21-bp deletion in the cytochrome B (CYTB) gene. This mutation was found in urine samples and cancer tissues from patients with bladder cancer [27]. Overexpression of the 21-bp deletion mutation of the *CYTB* gene induces rapid cell cycle progression through upregulation of the nuclear factor-kappa B2 signaling pathway and eventually leads to tumor growth in vivo and in vitro [29]. Additionally, mtDNA mutations in the electron transport chain (ETC) have been reported. Mutations in the NADH dehydrogenase subunit 4 (ND4) gene have been identified in UTUC. Approximately 85% of mutated *ND4* exists before the development of UTUC [30].

### 2.2. MtDNA Copy Number in UC

MtDNA copy number has been examined in bladder cancer and adjacent normal tissues using next-generation DNA sequencing. Compared with cells from normal tissues, bladder cancer cells were found to have lower mtDNA content. However, this reduction in mtDNA copy number was not accompanied by a reduction in mitochondrial gene expression. This discrepancy suggests that the expression of mitochondrial genes is not always correlated with mtDNA copy number and that mitochondrial activity may not be suppressed in bladder cancer [31].

### 2.3. Impact of Altered Expression of Mitochondrial Proteins on UC

Lon protease is an ATP-dependent serine protease in the mitochondrial matrix that is responsible for the degradation of abnormal proteins and maintenance of the mitochondrial genome. In cancers, Lon protease is essential for the proliferation and survival of cancer cells. Lon upregulation also contributes to metabolic reprogramming, facilitating the switch from respiratory to glycolytic metabolism in the cancer microenvironment [32]. In patients with bladder cancer, Lon protease expression is substantially higher in cancerous tissue than in non-cancerous tissue and is directly related to cancer grade and stage [33]. The mitochondrial GTPase mitofusin-2 (MFN2) is the key regulator of mitochondrial fusion at the outer mitochondrial membrane. Mitochondrial fusion/fission machinery plays a crucial role in mitochondrial quality control. Changes in mitochondrial fusion/fission machinery have been demonstrated in an increasing number of diseases including cancer [34,35]. The downregulation of MFN2 expression has been demonstrated in bladder cancer. In bladder cancer cell lines, MFN2 overexpression has been shown to inhibit cell proliferation by arresting the cell cycle and inducing apoptosis via caspase-3 [36]. Mitochondrial transcription factor A (TFAM) is a mitochondrial protein required for mtDNA stability, transcription, and replication [37,38]. TFAM expression was significantly enhanced in bladder cancer cells and directly related to cancer stage. In vitro studies have shown TFAM to induce bladder cancer cell proliferation, migration, and colony formation [39]. Leucine-rich pentatricopeptide repeat motif-containing protein (LRPPRC) is a multifunctional protein localized to the mitochondria, endoplasmic reticulum, outer and inner nuclear membranes, nucleoplasm, and cytoskeleton [40,41]. Besides being a prognostic factor, LRPPRC has recently been demonstrated to enhance tumorigenesis in bladder cancer [42].

The mitochondrial fusion/fission machinery is regulated by other genes. MicroRNA-98 (miR-98) regulates this fusion/fission machinery and affects mitochondrial membrane potential (MMP) in cancers. MiR-98 is known to be upregulated in bladder cancer cell lines and promote proliferation [43]. The role of miR-98 in chemoresistance depends on longevity assurance homolog 2 of yeast LAG1 (LASS2). LASS2 is a potent tumor suppressor that induces mitochondrial fusion and inhibits MMP. LASS2 consumption may lead to the proliferation and invasion of bladder cancer cells [44], and LASS2 negativity is associated with poor prognosis in bladder cancer [45].

### 2.4. Mitochondria Regulate Energy Metabolism in UC

**Figure 1:**
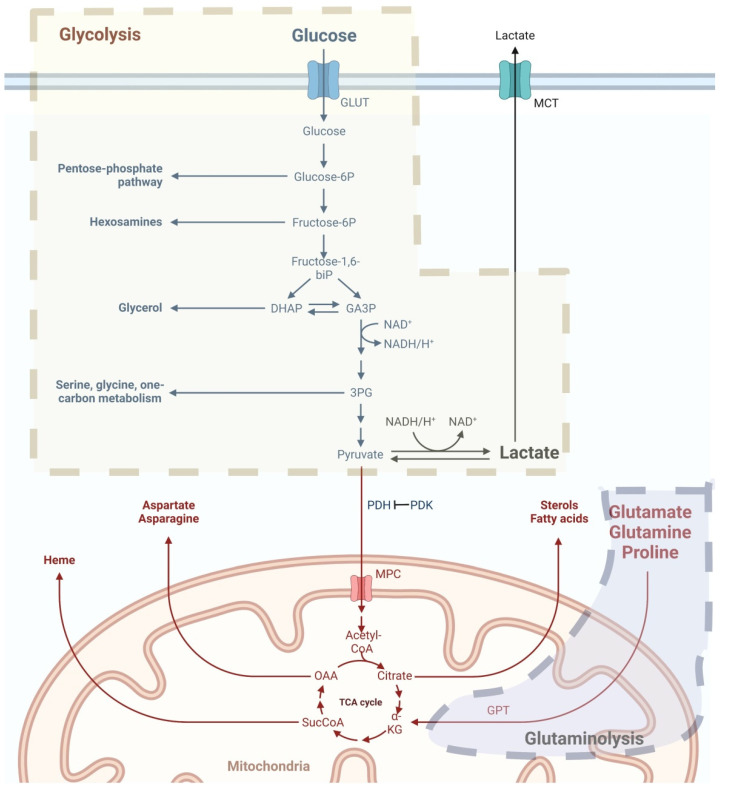
Mitochondria regulate metabolic reprogramming in UC. The major pathways of metabolic reprogramming in UC are enhanced aerobic glycolysis and glutaminolysis. Aerobic glycolysis leads to increased cytosolic lactate production. Glutaminolysis supports cancer cells by providing energy and pools of TCA cycle intermediates for biosynthesis of proteins, lipids, and nucleotides Abbreviations: GLUT, glucose transporter; GPT, glutamate pyruvate transaminase; MCT, monocarboxylate transporter; OAA, oxaloacetate; PDH, pyruvate dehydrogenase; PDK, pyruvate dehydrogenase kinases; TCA, tricarboxylic acid. The figure was created with BioRender.com.

Mitochondria participate in the metabolic reprogramming of cancer cells (Figure 1). The Warburg effect, which was first reported by Otto Warburg in 1926, describes that tumor cells uptake substantial glucose and undergo glycolysis as an energy supplement, even with sufficient oxygen. Aerobic glycolysis results in increased production of cytosolic lactate [46]. Non-neoplastic cells produce energy by glucose oxidation via mitochondria, which oxidizes pyruvate to acetyl-co-enzyme-A under aerobic conditions. In this situation, pyruvate dehydrogenase (PDH) enables pyruvate to enter mitochondria. Carcinogenesis is preferred in hypoxic tissues because glucose consumption is low. Hypoxia-inducible factor (HIF) 1α is then activated together with upregulated glucose transporters (GLUTs) and pyruvate dehydrogenase kinase (PDK). The activation of PDK leads to the inhibition of PDH, and thus, the inhibition of glycolysis. In UC, PDK3 overexpression has recently been linked to poor oncological outcomes. Together with the overexpression of PDK3, these co-upregulated genes were associated with DNA repair and replication. These results suggest that PDK3 plays a crucial role in the development and proliferation of UC [47].

The mitochondrial matrix hosts the tricarboxylic acid (TCA) cycle. In UC, the mitochondrial TCA cycle produces reducing equivalents to fuel ETC and generate biosynthetic intermediates that are necessary for cell proliferation [48,49]. In addition to lactate, other substrates, including glutamine, are known to fuel the TCA cycle and participate in energy production when coupled with oxidative phosphorylations [50,51,52]. By interacting with heterogeneous nuclear ribonucleoprotein (hnRNP) I/L to upregulate glutamate pyruvate transaminase (GPT2) expression, long non-coding RNA urothelial cancer associated 1 (UCA1) has recently been demonstrated to promote glutamine-driven anaplerosis in bladder cancer [53].

### 2.5. Altered Mitochondrial ROS Production and ETC Activity in UC

**Figure 2:**
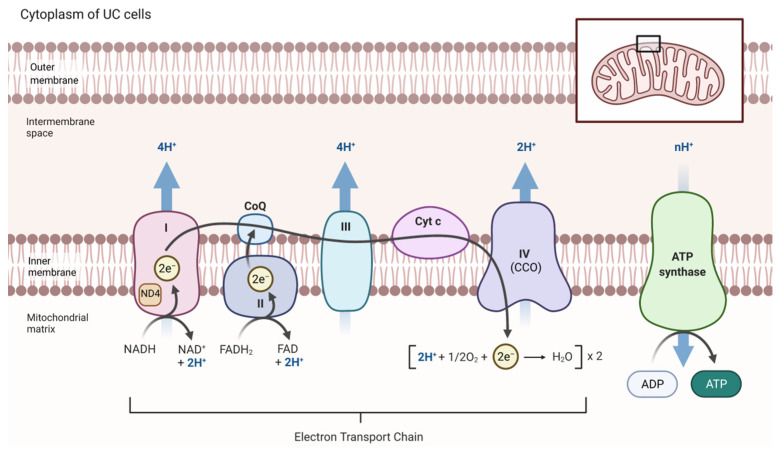
Schematic diagram of ETC in UC. ETC is located on the inner mitochondrial membrane and composed of five protein complexes. Mutations in the ND4 subunit of Complex I are found in UC. Complex IV is associated with progression and tumorigenesis UC. Abbreviations: CCO, cytochrome c oxidase; ND4, NADH dehydrogenase subunit 4. The figure was created with BioRender.com.

Redox homeostasis is a crucial mechanism in the progression and development of cancers [54]. Mitochondria generate ROS, which serve as toxic species for cellular macromolecules and regulate metabolic pathways [48]. Mitochondrial ROS are produced at the ETC by the leakage of electrons at the ubiquinone-binding sites of Complex I and Complex III [18,55]. Increased levels of ROS are related to increased metabolic activities and altered antioxidant capacities, which are often found in malignant conditions and interact with tumor growth and expansion [26,56]. Huang et al. investigated the urinary bladder of Sprague–Dawley rats after administering N-butyl-N-(4-hydroxybutyl) nitrosamine (BBN), a carcinogen, for eight weeks to evaluate tumorigenesis [57]. They measured the activities of components of the ETC, including NADH cytochrome c reductase (NCCR, Complex I+III), succinate cytochrome c reductase (SCCR, Complex II+III), and cytochrome c oxidase (CCO, Complex IV). The activities of all the NCCR, SCCR, and CCO were elevated by exposure to BBN, indicating a positive correlation with tumorigenesis. However, NCCR and SCCR activities reduced rapidly when BBN was discontinued, whereas CCO activity plateaued at 18 weeks despite the withdrawal of BBN. These results demonstrated that, compared with NCCR and SCCR, the CCO enzyme is more relevant to the progression of tumorigenesis in bladder cancer [57]. Figure 2 depicts the ETC in UC to facilitate the understanding of the altered ETC activity discussed above.

The majority of intracellular ROS are produced by mitochondria. Although the sources of ROS have not been specified in some studies, a few proteins have been reported to increase ROS generation in UC. The expression of membrane-associated leukotriene B4 receptor 2 (LTB4R2) is upregulated during the progression of bladder cancer. LTB4R2 enhances the expression of NADPH oxidase-1 and -4 (NOX-1 and NOX-4), which are members of the NADPH oxidase family known to generate ROS. The increased production of ROS and the activation of NF-κB further promote the invasion and metastasis of bladder cancer both in vivo and in vitro [58,59]. In addition, human alkylated DNA repair protein alkB homolog 8 (ALKBH8) is reported to be associated with the tumorigenesis of bladder cancer. In in vitro studies, silencing of ALKBH8 reduced ROS production via downregulation of NOX-1 and induced apoptosis via subsequent activation of p38 and c-Jun NH(2)-terminal kinase (JNK) [60].

### 2.6. Mitochondria Regulate Cell Death in UC

**Figure 3:**
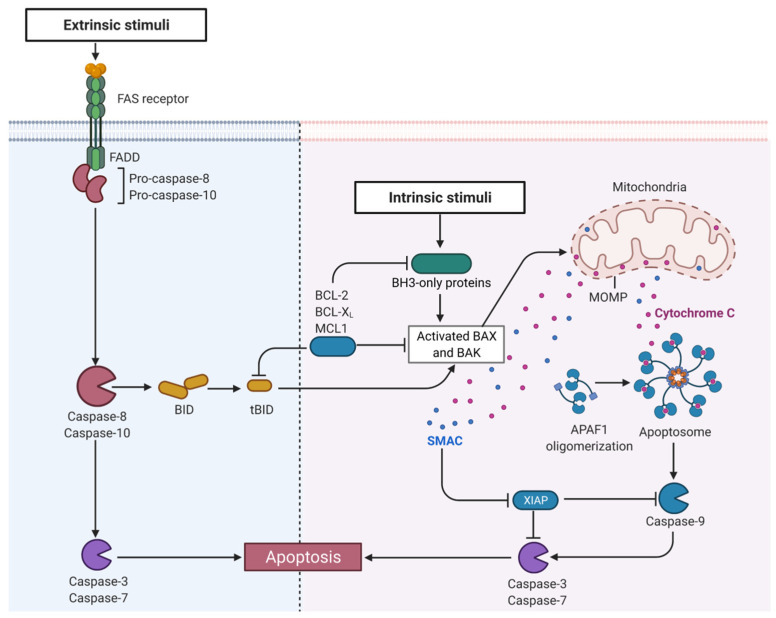
Mitochondria regulate apoptosis in UC. Abbreviations: APAF1, apoptotic protease-activating factor 1; BAK, Bcl-2 homologous antagonist/killer; BAX, Bcl-2-associated X protein; BCL, B-cell lymphoma; BH3, Bcl-2 homology domain 3; BID, BH3 interacting domain death agonist; FADD, Fas-associated via death domain; MCL1, myeloid leukemia cell differentiation protein-1; MOMP, mitochondrial outer membrane permeabilization; SMAC, the second mitochondria-derived activator of caspases; XIAP, X-linked inhibitor of apoptosis. The figure was created with BioRender.com.

Mitochondria are involved in apoptosis, necrosis, and necroptosis [48]. Proteins of the B-cell lymphoma-2 (BCL-2) family bind voltage-dependent anion channels to accelerate the release of cytochrome c and induce apoptosis [61]. Myeloid leukemia cell differentiation protein-1 (MCL1) and BCL-xL are found in various mitochondrial subcompartments and unleash the antiapoptotic activities by competing with proapoptotic members of the BCL-2 family [48]. The BCL-2/BAX ratio is correlated with cytochrome c and apoptosis-inducing factors (AIFs), which determine the capability for mitochondria-mediated apoptosis [29]. The functional roles of BCL-2 in UC have also been studied. BCL-2 overexpression is associated with poor prognosis, early recurrence of bladder cancer [62,63,64], and resistance to gene therapy and chemotherapy [65,66]. In patients with bladder cancer receiving intravesical chemotherapy after tumor resection, early relapse can be observed in patients with a BCL-2/BAX ratio > 1 and a p53 gene mutation [62]. Patients with BCL-2-positive bladder cancer have significantly worse survival than those with BCL-2-negative tumors [63]. Recently, apoptotic protease-activating factor 1 (APAF1) in UC has been reported to be the direct target gene of miR-1270, which could induce apoptosis and enhance the cisplatin chemosensitivity of cancer cells [67]. In addition, in UC, the expression of X-linked inhibitor of apoptosis (XIAP) is higher at a later TMN stage [68]. The second mitochondria-derived activator of caspases (SMAC) competitively binds to XIAP, leading to the release of caspases and allowing the execution of apoptosis [69,70]. Figure 3 illustrates mitochondria-regulated apoptosis in UC.

### 2.7. Mitochondria Regulate Cell Proliferation in UC

A distinguished feature of cancers is their sustained cellular proliferation resulting from altered expression of constitutive telomerase that determines the maintenance of telomere length. It is known that telomerase reverse transcriptase (TERT) shuttles from the nucleus to the mitochondria upon oxidative stress, preserving mitochondrial functions and decreasing oxidative stress, thus protecting mtDNA and nuclear DNA (nDNA) from oxidative damage to avoid apoptosis [71,72]. In a recent report, mutations in the TERT promoter accounted for 84% of UC patients [73]. A meta-analysis further elucidated that bladder cancer patients carrying TERT promoter mutations have a greater risk of recurrence [74]. Using algorithmic inference from cross-sectional data, Hayashi et al. suggested that TERT promoter mutations play a role in the tumorigenesis of bladder cancer [75].

## 3. Therapeutic Strategies Targeting Mitochondria in UC

### 3.1. Targeting the TCA

Dichloroacetate (DCA) is a PDK inhibitor that can activate PDH, promote glucose oxidation, and further decrease tumor growth and angiogenesis. It has been demonstrated to decrease proliferation rates, increase pyruvate oxidation, and increase mitochondrial activity in UC [76]. Recently, metformin was shown to work synergistically with DCA to inhibit proliferation and reduce metabolic activity in a canine UC cell line [77].

Vitamin K2 has also been shown to exert anticancer effects. Recently, vitamin K2 was reported to promote glycolysis in UC cells by enhancing glucose consumption and lactate production and inhibiting the TCA cycle by reducing the amount of acetyl-CoA. This vitamin K2-induced metabolic stress triggers AMPK-dependent autophagic cell death in UC cells [78].

### 3.2. Restoring Mitochondria-Driven Apoptosis

Induction of apoptosis is a principal anticancer strategy used to eliminate cancer cells. Understanding apoptotic signaling pathways may assist in the discovery of novel therapeutic targets [79,80]. To date, three signaling mechanisms involving apoptosis have been discovered: the death-receptor-mediated extrinsic pathway [81], mitochondria-mediated intrinsic pathway [82], and endoplasmic reticulum (ER) stress-mediated pathway [83]. Mitochondria play an important role in apoptosis. AIF is the first caspase-independent cell death effector that interacts with DNA and induces nuclear condensation and DNA fragmentation. To explore novel and effective therapies for UC, a plethora of studies on the potential mechanisms of apoptosis have been performed.

Taking advantage of antisense oligodeoxynucleotides (AS-ODNs) to downregulate *BCL-2* can partially sensitize bladder cancers to cisplatin and radiotherapy [84,85]. Studies have shown that *BCL-2*, *BAX*, and *p53* contribute to drug sensitivity and apoptosis status and may help predict disease progression or recurrence [62,64]. In advanced bladder cancer, quantifying BCL-2 may help select target patients who may benefit from neoadjuvant chemotherapy [63]. For example, cisplatin is an important chemotherapeutic agent that is used to treat UC. Cisplatin induces apoptosis in a mitochondria-dependent and death-receptor-independent manner. BCL-2 overexpression inhibits cisplatin-induced BAX translocation and downstream events. Small interfering RNA (siRNA) targeting *BCL-2* may help reverse cisplatin resistance in bladder cancer [66]. Bolenz et al. studied the application of AS-ODNs targeting BCL-2 and BCL-xL and revealed an effective improvement in the cytotoxicity of chemotherapeutic agents, not merely cisplatin but also gemcitabine, mitomycin C, and paclitaxel. The combined treatment resulted in notably higher death rates in nearly all cell lines [85].

Silibinin, a natural flavonoid, inhibits the growth of UC cells and induces caspase-dependent and caspase-independent apoptosis, which is associated with disruption of MMP and selective release of AIF and cytochrome c from mitochondria. In addition to inducing apoptosis via caspase activation in human UC cells, silibinin has been proven to be an intravesical chemotherapy for the inhibition of carcinogenesis and the progression of bladder cancer [86]. Additionally, the orally-fed silibinin has been reported to prevent N-butyl-N-(4-hydroxybutyl) nitrosamine (OH-BBN)-induced bladder carcinogenesis in mice. Accumulating evidence indicates that silibinin is an effective agent for chemotherapy against bladder tumor cells [86,87,88,89], as well as prostate [90,91], breast [92,93], skin [94], colon [95], lung [96], and kidney [97,98].

Baicalein is a flavone derived from the herb Huangqin, which is used in traditional Chinese medicine as an anti-inflammatory agent [99]. It induces apoptosis through a mitochondria-dependent caspase activation pathway in bladder cancer cells [100]. Wu et al. demonstrated that baicalein inhibits bladder cancer proliferation and migration in a dose-dependent manner via the reduction of phosphorylated NF-κB and MMP-2/9 expression [101].

Resveratrol is a polyphenolic compound naturally found in peanuts, mulberries, and grapes. It is an ingredient of red wine and exerts cardio- and neuroprotective effects [102,103]. In in vitro studies of UC, resveratrol has been shown to disrupt the MMP, increase ROS production, reduce ATP concentrations, provoke the release of cytochrome c from mitochondria to the cytosol, activate caspase-9 and caspase-3, and eventually induce apoptosis in cancer cells [104,105].

CXC195 also induces apoptosis by activating JNK, DP5, and PUMA, inhibiting BCL-2 and BCL-xL, and consequently inducing mitochondrial- and caspase-dependent apoptosis [79]. CXC195 is a tetramethylpyrazine (TMP) analog that displays antioxidant activity and antiapoptotic effects by inhibiting NADPH oxidase and iNOS expression and regulating the PI3K-AKT-GSK3b pathway. CXC195 is thought to be a promising anticancer drug that inhibits cell proliferation and inflammatory responses in bladder cancer [79].

Tumor necrosis factor (TNF)-related apoptosis-inducing ligand (TRAIL; Apo-2L) is a member of the TNF family and has recently gained attention because of its ability to induce apoptosis in cancers [106]. TRAIL induces apoptosis through a caspase-dependent mechanism, which can be strengthened by the release of cytochrome c and the loss of MMP [107]. TRAIL is a potent antitumor agent in preclinical studies; however, it has some limitations in potency. Combining TRAIL with other agents may improve cancer cell responsiveness. Histone deacetylase inhibitors have been shown to modulate the sensitivity of TRAIL-resistant bladder cancer cells [106].

### 3.3. Targeting Mitochondrial Turnover

Mitochondrial fusion, fission, and mitophagy have been examined as potential anticancer targets. Dynasore is a GTPase inhibitor of dynamin-related protein 1 (DRP1) [108]. Inhibition of mitochondrial fission by dynasore suppresses cancer cell proliferation and induces apoptosis. It inhibits migration and/or invasion in various cancer cell lines, including the bladder cancer cell line [109]. Radiation therapy may also play a role in UC treatment by causing mitochondrial damage. Shea et al. used cultured MGH-U1 (human urinary bladder carcinoma) cells and treated them with doxycycline and long-wave ultraviolet A (UVA) radiation. The cells were found to have mitochondrial damage when the UVA dose reached 1 J/cm^2^ and above [110].

### 3.4. Targeting Other Mitochondrial Modulators

Some proteins can indirectly modulate the mitochondrial function. NBR1 (a neighbor of the BRCA1 gene, an autophagy cargo receptor) is overexpressed in human UC cells. Rapamycin targeting the mammalian target of rapamycin (mTOR) kinase can regulate autophagy and has therapeutic effects in patients with cancer. In NBR1-knockdown UC cells, sensitivity to rapamycin-associated apoptosis and mitochondrial defects was enhanced. Loss of NBR1 expression changes cellular responses to rapamycin, leading to impaired ATP homeostasis and increased ROS levels. Therefore, NBR1 may be a potential therapeutic target for treating UC [86]. Table 1 summarizes mitochondria-targeted therapies for UC.

## 4. Conclusions and Perspectives

UC is a common but complex disease. By reviewing the available literature, we revisited the pathogenic role of mitochondria in UC. The main mechanisms by which mitochondria participate in tumorigenesis and progression of UC include mtDNA mutations, altered expression of mitochondrial proteins, metabolic reprogramming, deregulated mitochondrial ROS production and ETC activity, and mitochondria-regulated proliferation and death in cancer cells. The interplay between these different mechanisms often exists and complicates the whole process. Therapeutic strategies targeting these mitochondria-centered mechanisms are promising. They could be complementary to the current treatment modalities, including surgery, chemotherapy, and immunotherapy. Notably, the evidence summarized in this review is largely based on in vitro and animal studies. Advanced and detailed in vivo studies are required to facilitate future clinical research and clinical trials.

## Figures and Tables

**Table 1:** Mitochondria-targeted therapies for UC.

Therapies	Strategies	Targets	References
DCA	inhibit PDK and activate PDH	mitochondrial TCA	[76]
vitamin K2	promote the glycolysis	mitochondrial TCA	[78]
AS-ODNs	improve drug sensitivity induce apoptosis	BCL-2, NRB1	[84,85]
siRNA	improve drug sensitivity induce apoptosis	BCL-2, NRB1	[66]
metformin	induce apoptosis	mitochondria	[77]
silibinin	induce apoptosis	mitochondria	[86]
baicalein	induce apoptosis	mitochondria	[100]
resveratrol	induce apoptosis	mitochondria	[104,105]
CXC195	induce apoptosis	TMP analog	[79]
TRAIL	induce apoptosis	mitochondria	[106]
UVA	damage mitochondria	mitochondria	[110]

AS-ODNs, antisense oligodeoxynucleotides; siRNA, small interfering RNA; TMP, tetramethylpyrazine; TRAIL, tumor necrosis factor (TNF)-related apoptosis-inducing ligand; DCA, dichloroacetate; PDK, pyruvate dehydrogenase kinase; PDH, pyruvate dehydrogenase; UVA, ultraviolet A.

## Data Availability

Not applicable.
